# Supplementary material for: DLLabelsCT: Annotation tool using deep transfer learning to assist in creating new datasets from abdominal computed tomography scans, case study: Pancreas
Source: PLoS One. 2024 Dec 3;19(12):e0313126. doi: 10.1371/journal.pone.0313126 (PMC11614254; doi:10.1371/journal.pone.0313126)

PLOS ONE

DLLabelsCT: Annotation tool using deep transfer learning to assist in creating new datasets from abdominal computed tomography scans, case study: pancreas

--Manuscript Draft--

**Manuscript Number:**	PONE-D-24-21753
**Article Type:**	Research Article
**Full Title:**	DLLabelsCT: Annotation tool using deep transfer learning to assist in creating new datasets from abdominal computed tomography scans, case study: pancreas
**Short Title:**	Annotation tool with deep learning assistant for abdominal computed tomography scans
**Corresponding Author:**	Henrik Mustonen<br>University of Oulu: Oulun Yliopisto<br>Oulu, FINLAND
**Keywords:**	Artificial intelligence; Deep Learning; transfer learning; abdominal computed tomography scan; annotation tool; pancreas segmentation; organ segmentation
**Abstract:**	The utilization of artificial intelligence (AI) is expanding significantly within medical research and, to some extent, in clinical practice. Deep learning (DL) applications, which use large convolutional neural networks (CNN), hold considerable potential, especially in optimizing radiological evaluations. However, training DL algorithms to clinical standards requires extensive datasets, and their processing is labor-intensive. In this study, we developed an annotation tool named DLLabelsCT that utilizes CNN models to accelerate the image analysis process. To validate DLLabelsCT, we trained a CNN model with a ResNet34 encoder and a UNet decoder to segment the pancreas on an open-access dataset and used the DL model to assist in annotating a local dataset, which was further used to refine the model. DLLabelsCT was also tested on two external testing datasets. The tool accelerates annotation by 3.4 times compared to a completely manual annotation method. Out of 3,715 CT scan slices in the testing datasets, 50% did not require editing when reviewing the segmentations made by the ResNet34-UNet model, and the mean and standard deviation of the Dice similarity coefficient was 0.82±0.24. DLLabelsCT is highly accurate and significantly saves time and resources. Furthermore, it can be easily modified to support other deep learning models for other organs, making it an efficient tool for future research involving larger datasets.
**Order of Authors:**	Henrik Mustonen, MSc (tech.)
	Antti Isosalo, MSc (tech.)
	Minna Nortunen, PhD
	Mika Nevalainen, PhD
	Miika Nieminen, Prof
	Heikki Huhta, PhD
**Additional Information:**	
**Question**	**Response**
**Financial Disclosure**<br><br>Enter a financial disclosure statement that describes the sources of funding for the work included in this submission. Review the submission guidelines for detailed requirements. View published research articles from *PLOS ONE* for specific examples.	The author(s) received no specific funding for this work.

Powered by Editorial Manager® and ProduXion Manager® from Aries Systems Corporation

This statement is required for submission and **will appear in the published article** if the submission is accepted. Please make sure it is accurate.<br><br>**Funded studies**<br>Enter a statement with the following details:<br>• Initials of the authors who received each award<br>• Grant numbers awarded to each author<br>• The full name of each funder<br>• URL of each funder website<br>• Did the sponsors or funders play any role in the study design, data collection and analysis, decision to publish, or preparation of the manuscript?<br><br>Did you receive funding for this work?	
**Competing Interests**<br><br>Use the instructions below to enter a competing interest statement for this submission. On behalf of all authors, disclose any competing interests that could be perceived to bias this work—acknowledging all financial support and any other relevant financial or non-financial competing interests.<br><br>This statement is required for submission and **will appear in the published article** if the submission is accepted. Please make sure it is accurate and that any funding sources listed in your Funding Information later in the submission form are also declared in your Financial Disclosure statement.<br><br>View published research articles from *PLOS ONE* for specific examples.	The authors have declared that no competing interests exist.

Powered by Editorial Manager® and ProduXion Manager® from Aries Systems Corporation

**NO authors have competing interests**<br><br>Enter: *The authors have declared that no competing interests exist.*<br><br>**Authors with competing interests**<br><br>Enter competing interest details beginning with this statement:<br><br>*I have read the journal's policy and the authors of this manuscript have the following competing interests: [insert competing interests here]*<br><br>* typeset	
**Ethics Statement**<br><br>Enter an ethics statement for this submission. This statement is required if the study involved:<br><br>• Human participants<br>• Human specimens or tissue<br>• Vertebrate animals or cephalopods<br>• Vertebrate embryos or tissues<br>• Field research<br><br>Write "N/A" if the submission does not require an ethics statement.<br><br>General guidance is provided below. Consult the submission guidelines for detailed instructions. **Make sure that all information entered here is included in the Methods section of the manuscript.**	This study complied with the ethical standards of the Oulu University Institutional Research Committee and the Declaration of Helsinki (as revised in 2013). Data collection from included hospitals was approved by the Finnish Social and Health Data Permit Authority (FINDATA, Dnro THL/3606/14.02.00/2020). Individual patient consent for this retrospective analysis was waived.

Powered by Editorial Manager® and ProduXion Manager® from Aries Systems Corporation

**Format for specific study types**<br><br>**Human Subject Research (involving human participants and/or tissue)**<br>• Give the name of the institutional review board or ethics committee that approved the study<br>• Include the approval number and/or a statement indicating approval of this research<br>• Indicate the form of consent obtained (written/oral) or the reason that consent was not obtained (e.g. the data were analyzed anonymously)<br><br>**Animal Research (involving vertebrate animals, embryos or tissues)**<br>• Provide the name of the Institutional Animal Care and Use Committee (IACUC) or other relevant ethics board that reviewed the study protocol, and indicate whether they approved this research or granted a formal waiver of ethical approval<br>• Include an approval number if one was obtained<br>• If the study involved *non-human primates*, add *additional details* about animal welfare and steps taken to ameliorate suffering<br>• If anesthesia, euthanasia, or any kind of animal sacrifice is part of the study, include briefly which substances and/or methods were applied<br><br>**Field Research**<br><br>Include the following details if this study involves the collection of plant, animal, or other materials from a natural setting:<br>• Field permit number<br>• Name of the institution or relevant body that granted permission	
**Data Availability**<br><br>Authors are required to make all data underlying the findings described fully available, without restriction, and from the time of publication. PLOS allows rare exceptions to address legal and ethical concerns. See the PLOS Data Policy and FAQ for detailed information.	No - some restrictions will apply

A Data Availability Statement describing where the data can be found is required at submission. Your answers to this question constitute the Data Availability Statement and **will be published in the article**, if accepted.<br><br>**Important:** Stating 'data available on request from the author' is not sufficient. If your data are only available upon request, select 'No' for the first question and explain your exceptional situation in the text box.<br><br>Do the authors confirm that all data underlying the findings described in their manuscript are fully available without restriction?	
**Describe where the data may be found in full sentences. If you are copying our sample text, replace any instances of XXX with the appropriate details.**<br><br>• If the data are **held or will be held in a public repository**, include URLs, accession numbers or DOIs. If this information will only be available after acceptance, indicate this by ticking the box below. For example: *All XXX files are available from the XXX database (accession number(s) XXX, XXX.)*.<br>• If the data are all contained **within the manuscript and/or Supporting Information files**, enter the following: *All relevant data are within the manuscript and its Supporting Information files.*<br>• If neither of these applies but you are able to provide **details of access elsewhere**, with or without limitations, please do so. For example:<br><br>*Data cannot be shared publicly because of [XXX]. Data are available from the XXX Institutional Data Access / Ethics Committee (contact via XXX) for researchers who meet the criteria for access to confidential data.*<br><br>*The data underlying the results presented in the study are available from (include the name of the third party*	The annotation tool can be found from https://github.com/MIPT-Oulu/DLLabelsCT Any patients related data is not available due to Finnish legislation

and contact information or URL).<br>• This text is appropriate if the data are owned by a third party and authors do not have permission to share the data.<br><br>* typeset	
Additional data availability information:	

Manuscript Click here to access/download;Manuscript;Manuscript.docx

DLLabelsCT: Annotation tool using deep transfer learning to assist in creating new datasets from abdominal computed tomography scans, case study: pancreas

Henrik Mustonen[2*], Antti Isosalo[2], Minna Nortunen[1,3], Mika Nevalainen[2,4], Miika Nieminen[2,4], Heikki Huhta[1,3]

[1]Research Unit of Translational Medicine, Oulu University Hospital, Oulu, Finland

[2]Research Unit of Health Sciences and Technology, Faculty of Medicine, University of Oulu, Oulu, Finland

[3]Department of Surgery, Oulu University Hospital, Oulu, Finland

[4]Department of Diagnostic Radiology, Oulu University Hospital, Oulu, Finland

* Corresponding author

E-mail: henrik.mustonen@oulu.fi

Abstract

The utilization of artificial intelligence (AI) is expanding significantly within medical research and, to some extent, in clinical practice. Deep learning (DL) applications, which use large convolutional neural networks (CNN), hold considerable potential, especially in optimizing radiological evaluations. However, training DL algorithms to clinical standards requires extensive datasets, and their processing is labor-intensive. In this study, we developed an annotation tool named DLLabelsCT that utilizes CNN models to accelerate the image analysis process. To validate DLLabelsCT, we trained a CNN model with a ResNet34 encoder and a UNet decoder to segment the pancreas on an open-access dataset and used the DL model to assist in annotating a local dataset, which was further used to refine the model. DLLabelsCT was also tested on two external testing datasets. The tool accelerates annotation by 3.4 times compared to a completely manual annotation method. Out of 3,715 CT scan slices in the testing datasets, 50% did not require editing when reviewing the segmentations made by the ResNet34-UNet model, and the mean and standard deviation of the Dice similarity coefficient was 0.82±0.24. DLLabelsCT is highly accurate and significantly saves time and resources. Furthermore, it can be easily modified to support other deep learning models for other organs, making it an efficient tool for future research involving larger datasets.

Introduction

Artificial intelligence (AI) has shown promising results in the field of medical research and medical image analysis[1]. Deep learning (DL) is a subset of AI that involves the use of artificial neural networks to learn and recognize patterns in data, such as computed tomography (CT) and magnetic resonance imaging (MRI) scans, which can provide detailed information about the abdominal organs or location and characteristics of tumors[2]. To enhance the specificity and sensitivity of the DL algorithms for instance in cancer detection, it is crucial to have access to high-quality data representing different cancer types and stages[2]. This requires large datasets of medical images, which are annotated by expert radiologists, vital for DL algorithms to learn accurately. The task is time-consuming and costly[2]. Annotation tools typically provide semi-automated segmentation methods, which are either based on previous annotations requiring input from the user, like selecting the region for the automatic segmentation method[3]. Fully automated segmentation methods do not necessarily require user input and with large datasets it would improve efficiency and be beneficial.

Fully automated methods are the most effective when the segmented object differs heavily from its surroundings[4]. In CT scans the surrounding organs have similar Hounsfield Unit (HU) -values, making automatic segmenting more difficult[4]. DL-based segmentation methods have proven very accurate for segmenting organs in abdominal CT scans[4]. There are only a few validated annotation tools that support DL-methods, such as RIL-Contour[5]. Training of DL algorithms to clinical standards requires extensive datasets, and their processing is exceedingly time-consuming. Tools that automatically annotate organs or tumors from CT images can expedite the management of these large datasets. Yet, the availability of validated and published DL-based annotation tools remains limited.

The purpose of this study was to evaluate the efficacy of our newly developed deep learning-based annotation tool, named DLLabelsCT (Deep Learning Labels Computed Tomography), in accelerating the annotation process of organs in CT scans versus traditional manual annotation. DLLabelsCT is available from https://zenodo.org/records/10226990. The pancreas was selected as the organ for annotation, since its segmentation is a difficult task, due to the varying of the parenchymal shape, density, contrast enhancement and size within the abdominal CT-scan[6] making it a more challenging object for DL compared, for instance, to the liver.

Materials

Four independent CT scan datasets were used. The CT imaging in these datasets was performed in the portal venous phase and the image size was 512-by-512 pixels with varying pixel spacings. The scans were originally in Digital Imaging and Communications in Medicine (DICOM) format. Windowing was performed on DICOM-images, with the window width w_l of 400 HU and the window center w_c of 50 HU, for improved contrast between the tissue types. Windowing sets the images output values based on the following equation,

$$y = \left(\frac{x-(w_c-0.5)}{w_l-1} + 0.5\right) \cdot (y_{max} - y_{min}) + y_{min} \quad (1)$$

where x is the input value, y is the output value and y_{min} and y_{max} are the minimum and maximum possible outputs for the image format, i.e. for 16-bit images the values are 0 and 65535. Values outside the window were set to the maximum and minimum. The individual slices were then transformed into 16-bit PNG images from the DICOM format. The PNG images were then used to train the convolutional neural network (CNN) model.

Datasets

Training dataset

To train the DL-based segmentation method described in this study, an open-access National Institutes of Health Clinical Center dataset of contrast enhanced CT scans from the Cancer Imaging Archive was utilized[7,8,9]. The dataset consisted of 80 abdominal CT scans from healthy subjects with annotations for the pancreas. The dataset is referred to as Pancreas-CT from hereafter. The dataset contained a total of 18,942 axial slices and the slice thickness of the scans was 1.5-2.5 mm. This dataset was divided into training and testing datasets, with the testing dataset containing 20% of the scans. The patients were randomly split into the datasets without any stratification. According to the Cancer Imaging Archive, the volumes were acquired using CT scanning systems by Philips (Amsterdam, Netherlands) and Siemens (Erlangen, Germany). The X-ray tube voltages of the scanners were 120 kVp. This dataset was initially downloaded in November 2022.

Validation dataset

A dataset from the Oulu University Hospital was used to further train the segmentation model and to validate our novel in-house annotation tool. We refer to this dataset later as the Oulu validation dataset. The Oulu validation dataset contained CT scans from 606 patients, 313 with healthy pancreas, 218 with cancerous pancreas and 75 with other pancreatic diseases, such as intraductal pancreatic mucinous neoplasms, chronic pancreatitis etc. The slice thickness in Oulu validation dataset's scans varied between 0.625–7 mm. These scans contained a total of 96,429 axial slices. The CT scanners were manufactured by Toshiba (Minato, Tokyo, Japan), Philips (Amsterdam, Netherlands), Canon Medical Systems (Otawara, Tochigi, Japan), Siemens (Erlangen, Germany) and GE Medical Systems (Chicago, Illinois, United States). Tube voltages ranged between 80–140 kVp. This dataset was accessed and annotated between January and March 2023.

Testing datasets

Two accessory datasets from the Kuopio University Hospital (CT scans of 56 patients) and from Turku University Hospital (CT scans of eight patients) were combined to form the external testing dataset for accessing the performance of the annotation tools. All CT scans were of patients with pancreatic cancer. The dataset had a total of 26,154 axial slices, of which 12,227 were in the wanted portal venous phase. The slice thickness in these scans was 1–5 mm. The CT scanners were made by Toshiba (Minato, Tokyo, Japan), GE Medical Systems (Chicago, Illinois, United States) and Siemens (Erlangen, Germany). Tube voltages were 80–120 kVp. These datasets were accessed and annotated between April and May 2023.

The second testing dataset was from the Oulu University Hospital, containing 70 CT-scans with pancreatic cancer and which had not been previously used in the training dataset. We refer to this dataset later as the Oulu testing dataset. This dataset had a total of 17,558 axial slices with slice thickness of 0.5–5 mm. The CT scanners were made by Toshiba (Minato, Tokyo, Japan), GE Medical Systems (Chicago, Illinois, United States), Siemens (Erlangen, Germany), Philips (Amsterdam, Netherlands) and Canon Medical Systems (Otawara, Tochigi, Japan). Tube voltages were 80–120 kVp. The validation datasets were divided into two, with each half annotated by using a different tool (see chapters *Annotation tools* and *Method evaluation)*. This testing dataset was

accessed and annotated after the Oulu validation dataset, between March and April 2023.

Methods

Deep learning model

A 2-D CNN model with an encoder-decoder architecture was trained and used to segment pancreas from the CT-scans. The architecture utilizes a downsampling encoder that provides features for the upsampling decoder, which provides a prediction for each pixel in the image (Figure 1). The model employs a ResNet34[10] encoder that had been pretrained on the ImageNet[11] dataset, combined with a U–Net[12] decoder with randomly initialized weights. We refer to the model later as ResNet34UNet.

Fig 1: Example of how the Resnet34UNet model functions.

Model training

Initial training of the ResNet34UNet model was conducted on the open Pancreas-CT dataset. The training was implemented in a fully supervised manner by using the pixel-accurate annotations provided with the dataset. The training was performed on the training part of the Pancreas-CT dataset, including slices that did not contain pancreas.

Five-fold-cross-validation with non-interlapping groups was used, with the groups generated with Scikit-learn's[13] GroupKFold function. The model was trained for 50 epochs with a batch size of 16. The loss function utilized during training was Focal Tversky loss (FT)[14,15], defined as

$$\mathrm{FT} = \left(\frac{TP+1}{TP+\alpha\cdot FP+\beta\cdot FN+1}\right)^{\gamma} \quad (2)$$

where the α parameter is 0.7, β is 0.3 and γ is 0.75. Multiple data augmentations were used during training (Table 1). The augmentations were retrieved from the Streaming Over Lightweight Transformations (SOLT)[16] library version 0.1.8 and each of the augmentations had a 50% chance of occurring. Adam[17] was used as the optimizer during the training, with a multi-step learning rate scheduler. The initial learning rate and weight decay were set at 10^{-4}. The learning rate was reduced by a factor of 0.1 after 20, 30 and 40 epochs. The computer used had a Nvidia GeForce RTX 3090 graphics processing unit (GPU), an AMD Ryzen 9 5950 16-core central processing unit (CPU)

and 64 GB of 2400 MHz random-access memory (RAM). The computer's OS was Ubuntu 20.04.1 and the used Python version was 3.10.10 with PyTorch 1.13.1 and CUDA version 11.6. With this hardware the initial training took 24 hours.

Table 1. Data augmentations used on the data during model training.

Augmentation	Parameters [units]
Rotation	[-5, 5] [rad]
Scale	[0.6, 1.4] [au]
Translation	80 [px]
Random crop	448 × 448 [px]
Horizontal and vertical flip	
Gamma correction	[0.5, 1.8] [au]
Brightness and contrast	[30, 100] [au]
Salt and pepper noise	0.1 [au]
Gaussian noise	0.5 [au]
Gaussian blur (kernel sizes, sigma)	(3, 7, 11), (1, 10)
Median blur (kernel sizes, sigma)	(3, 7, 11), (1, 10)
Cutout	20%

Rad: radian, au:arbitrary unit, px: pixels

After training the ResNet34UNet model, it was used to provide initial annotations for the Oulu validation dataset. These initial annotations were then reviewed by our medical professionals (H.H, M.N and M.T.N). This checked segmented dataset was then combined with the Pancreas-CT dataset to train a new ResNet34Unet model, using the same training arguments as the initial model. To reduce training time, the number of images in the combined dataset was reduced by removing slices not containing pancreas. The training dataset contained 31,634 slices.

Annotation tools

Two different annotation tools were used and compared in the study. Our novel in-house tool DLLabelsCT was developed for the current study using Python (version 3.10.10) , which uses DL to assist in annotating (Figure 2). Additionally a previously developed MATLAB-based (2020a, Natick, MA, United States) tool named MammogramAnnotationTool[18] without any intelligent features (Figure 3) was used.

This tool was modified to support CT scans and was initially used in annotating the datasets, the modified tool was renamed CTAnnotationTool. DLLabelsCT was developed to speed up the annotation process. DLLabelsCT combines segmenting CT scans with a PyTorch based CNN and annotating the images manually. It uses a PyQt (2022, Dorchester, United Kingdom) based interface for the annotating. PyQt was selected as the base since it uses Python, as does PyTorch, and it is simple to use and understand. DLLabelsCT saves the annotations and the individual axial slices as PNG images, which can then directly be used as data in our DL model's training pipeline. The tool supports segmentation models with ResNet encoders and UNet or Feature Pyramid Network (FPN)[19] decoders.

Fig 2: DLLabelsCT with an example scan from the Pancreas-CT dataset. The shown masks can be modified, and new labels added from the menu on the right.

Fig 3: MATLAB-based annotation tool graphical user interface with an example scan from the Pancreas-CT dataset. The study can be changed from the window on the left and labels can be added on the CT scan on the window to the right.

Method evaluation

The external testing dataset and the Oulu testing dataset were used for testing the accelerating effect of our fully automated segmentation tool. Experienced, independent, and mutually blinded pancreatic surgeons (H.H for external and M.N. for Oulu) provided annotations using both the conventional CTAnnotationTool and the novel fully automated DLLabelsCT. The CTAnnotationTool was used to annotate 32 patients' pancreas from the external testing dataset and 31 patients from the Oulu testing dataset. The rest of the patients, 32 from the external testing dataset and 39 from the Oulu testing dataset, were annotated using DLLabelsCT with the initial DL model segmentations. Initial segmentations review was done with similar method between reviewers; segmentation needs to fill the entire pancreas and if not, it was corrected. The number of annotated slices and the time required for annotation were recorded for all patients. Additionally, we assessed the extent of revisions needed to the segmentations made by the ResNet34Unet model. The Dice similarity coefficient (DSC), defined as

$$\mathrm{DSC} = \frac{2TP}{2TP+FP+FN} \tag{3}$$

was calculated between the boolean masks made by the model and the segmentations modified with the fully automated segmentation tool.

Ethical statement

This study complied with the ethical standards of the Oulu University Institutional Research Committee and the Declaration of Helsinki (as revised in 2013). Data collection from included hospitals was approved by the Finnish Social and Health Data Permit Authority (FINDATA, Dnro THL/3606/14.02.00/2020). Individual patient consent for this retrospective analysis was waived. Additionally, ethical permission from the local wellbeing services county (Pohde) and ethical permission EETMK: 81 / 2008 were received.

Results

ResNet34UNet model trained on the training subset of Pancreas-CT had lower segmentation accuracies than the ResNet34UNet model trained on the Oulu validation dataset and the training subset of Pancreas-CT (Table 2, Figure 4). The segmentation accuracy was higher on the external testing dataset than the Oulu testing dataset (Table 2). The pancreas detection results (whether the model's segmentation mask and the final annotation mask overlap) for each individual slice in the CT scan were high for both of the models, with the ResNet34UNet model trained on the training subset of Pancreas-CT and the Oulu validation dataset having the higher accuracy on the testing subset of Pancreas-CT, and the accuracy was higher on the external testing dataset than the Oulu testing dataset (Table 3).

Table 2: Segmentation results of the ResNet34UNet model trained on the Pancreas-CT dataset (Model 1) and the ResNet34UNet model trained on Pancreas-CT and Oulu validation dataset (Model 2). Model 2 was used in segmenting the external testing dataset and Oulu testing dataset. Results presented as mean±standard deviation.

	Model 1 on Pancreas-CT	Model 2 on Pancreas-CT	External testing dataset	Oulu testing dataset
DSC	0.79±0.14	0.82±0.14	0.89±0.21	0.80±0.24
Recall	0.75±0.18	0.80±0.17	0.89±0.23	0.80±0.27
Precision	0.88±0.13	0.87±0.13	0.94±0.17	0.84±0.22

DSC: Dice similarity coefficient

Table 3: Pancreas detection (whether the ground truth mask and the model segmentation overlap on a CT scan slice) results of the ResNet34UNet model trained on the Pancreas-CT dataset (Model 1) and the ResNet34UNet model trained on Pancreas-CT and Oulu validation dataset (Model 2). Model 2 was used in segmenting the external testing dataset and Oulu testing dataset. Results presented as mean±standard deviation.

	Model 1 on Pancreas-CT	Model 2 on Pancreas-CT	External testing dataset	Oulu testing dataset
Accuracy	95.2%	95.4%	97.6%	96.5%
Specificity	98.8%	96.3%	98.3%	98.9%
Sensitivity	88.9%	93.8%	94.9%	88.3%
PPV	97.7%	93.6%	94.1%	96.0%
NPV	93.9%	96.4%	98.6%	96.6%

PPV: Positive predictive value, NPV: Negative predictive value

Fig 4: Example result on a CT scan from the Pancreas-CT dataset. Model 1 is the ResNet34UNet model trained on the training subset of the Pancreas-CT dataset and Model 2 is the ResNet34UNet model trained on both the training subset of the Pancreas-CT dataset and Oulu validation dataset.

Annotating the datasets was faster with DLLabelsCT than with CTAnnotationTool, even though the dataset annotated with DLLabelsCT contained more slices (Table 4, Table 5). The ResNet34UNet model's segmentations made annotating 3.4 times faster on average (Table 4, Table 5). In the external testing dataset 62% of the model segmentations were accepted without revision, while 44% of the segmentations in Oulu testing dataset needed no adjusting. In total 50% of the segmentations did not need adjusting.

Table 4: Annotation times for the external testing dataset

	Python DLLabelsCT	MATLAB CTAnnotationTool
Number of scans	32	32
Total time (seconds)	3223	8486
Total slices	1313	926
Time per slice (seconds)	2.5	9.2
Average time per scan (seconds)	94.8	73.0
Average number of slices in a scan	38.6	27.2

Table 5: Annotation times for the Oulu testing dataset

	Python DLLabelsCT	MATLAB CTAnnotationTool
Number of scans	39	31
Total time (seconds)	10304	12287
Total slices	2402	884
Time per slice (seconds)	4.3	13.9
Average time per scan (seconds)	264.2	396.4
Average number of slices in a scan	61.6	28.5

Discussion

The current study demonstrates that it is possible to create a highly accurate DL-based fully automated segmentation tool for annotating organs from abdominal CT scans with a relatively small amount of data. Furthermore, we show that the use of fully automated software saves a significant amount of time, making it cost-effective.

The used DL segmentation method has higher DSC than the initial method proposed with the Pancreas-CT dataset[6], with the ResNet34UNet model having a mean DSC of 0.82 over the mean DSC of 0.72 of the method proposed in the previously mentioned article. Newer DL-based segmentation methods have achieved a larger DSC than our method, with a DL segmentation method achieving a mean DSC of 0.90 on the Pancreas-CT dataset[20]. The DL method used in our study is more of an example of the

potential of DL and DLLabelsCT can be easily modified to support different PyTorch-based segmentation models.

DL neural networks are under intensive research and have a wide range of possible applications also in the field of medical research. For instance, genomics, metagenomics, histological and radiological image recognition benefit from AI evolution[20]. Recently various studies have shown promising results from DL network for pancreas segmentation. However, prior DL-based pancreatic segmentation studies utilized the Pancreas-CT dataset (n = 82). While the previously suggested DL networks attained commendable performance for pancreas segmentation (mean DSC of 0.866 and 0.854), the available data remains insufficient to establish the reliability of these networks since DL-based medical image segmentation is highly dependent on the number of data points[22,23].

To the best of our knowledge, the only study conducted with a relatively large amount of data (1006 patients) for normal healthy pancreatic segmentation is the Korean study, whose results closely aligned with our own, DSC (0.84 and our 0.82)[24]. It's important to note, however, that our dataset included 43% of patients with pathological pancreases. Additionally, our segmentation method for the pancreas achieved a DSC of 0.80 within the Oulu testing dataset, which exclusively comprises patients diagnosed with pancreatic ductal adenocarcinoma which is significantly impacting to pancreas normal shape, size and volume of pancreatic parenchyma[25]. Previous studies have shown more modest performance in similar conditions[26]. Promising results have also been achieved using neural networks for cancer detection and disease prognosis classification[21,27]. Using CNN, small pancreatic cancers with a diameter of less than 2cm were identified from CT images with the same or even better accuracy than radiology specialists[28].

DL methods can give more reliable outputs than other segmentation methods, such as active contours, region growing, histogram-based methods etc. since the DL models learn the varying shapes and contrast of the intended target[29]. DL methods do not require any additional input from the user and the initial annotation masks can be created with no imminent user. The disadvantages of DL are the long training time and the large computational requirements.

Developing DL applications for pre-operative assessment for instance, of pancreatic tumors requires large patient cohorts to capture disease variations[30]. Challenges in developing algorithms from large nation-wide datasets include varying imaging

equipment, contrast agent concentrations, and slice thicknesses[31]. The algorithm must also learn patient-specific factors such as comorbidities, age, body composition, and circulatory issues to function effectively[31]. Given resource limitations, manual annotation of massive datasets is unfeasible. Automated DL annotation tools like DLLabelsCT are necessary for processing these datasets. Validation, reporting, and publication of these tools are crucial for assessing the quality and reliability of the data processed. In our study, the in-house tool DLLabelsCT was 3.4 times faster than manual annotation, even with pancreatic cancer cases.

There are some limitations to this study. First, the small amount of CT scans used in evaluating differences between the annotation tools causes bias and could hamper the comparison. This was compensated for by having data from multiple sources with different types of data, which contributes to a better generalizability of the developed model. Similarly, different data from various CT scanners and sources were used in training the DL model. A technical limitation arises from using DL with a reasonable speed which requires a compatible GPU. Nonetheless, DLLabelsCT can be used without a GPU and the model's segmentation masks can be provided from a separate computer with a GPU.

In conclusion, the results demonstrate that our DL based fully automated segmentation and annotation tool DLLabelsCT for pancreas is highly accurate and saves time and resources significantly. Moreover, it could easily modify to detect other organs as well with small data amount and will be an efficient tool for future research with larger datasets. The annotation tool is publicly available at https://github.com/MIPT-Oulu/DLLabelsCT.

Data availability

Original CT scan data from the Finnish hospitals is not available due to Finnish legislation concerning the sharing of patients' related data. The open Pancreas-CT dataset is available from The Cancer Imaging Archive https://wiki.cancerimagingarchive.net/display/public/pancreas-ct

Acknowledgements:

Special thanks: Esa Liukkonen and Anne Kukkonen for their excellent technical assistance on data facilitation

References

1. Montagnon E, Cerny M, Cadrin-Chênevert A, Hamilton V, Derennes T, Ilinca A, et al. Deep learning workflow in radiology: a primer. Insights Imaging. 2020 Dec;11(22).

2. Mazurowski MA, Buda M, Saha A, Bashir MR. Deep learning in radiology: An overview of the concepts and a survey of the state of the art with focus on MRI. Magnetic Resonance Imaging. 2019 Apr;49(4):939-54.

3. Aljabri M, AlAmir M, AlGhamdi M, Abdel-Mottaleb M, Collado-Mesa F. Towards a better understanding of annotation tools for medical imaging: a survey. Multimed Tools Appl. 2022 Jul;81(18):25877-911.

4. Gibson E, Giganti F, Hu Y, Bonmati E, Bandula S, Gurusamy K, et al. Automatic Multi-Organ Segmentation on Abdominal CT With Dense V-Networks. IEEE Trans Med Imaging. 2018 Aug;37(8):1822-34.

5. Philbrick KA, Weston AD, Akkus Z, Kline TL, Korfiatis P, Sakinis T, et al. RIL-Contour: a Medical Imaging Dataset Annotation Tool for and with Deep Learning. J Digit Imaging. 2019 Aug;32(4):571-81.

6. Roth H, Farag A, Lu L, Turkbey EB, Summers RM. Deep convolutional networks for pancreas segmentation in CT imaging. Proc. SPIE 9413, Medical Imaging 2015: Image Processing, 94131G, DOI:10.1117/12.2081420.

7. Roth H, Farag A, Turkbey EB, Lu L, Liu J, Summers RM. 2016. Data From Pancreas-CT (Version 2) [Data set]. The Cancer Imaging Archive. https://doi.org/10.7937/K9/TCIA.2016.tNB1kqBU

8. Roth H, Lu L, Farag A, Shin H, Liu J, Turkbey EB, et al. DeepOrgan: Multi-level deep convolutional networks for automated pancreas segmentation. Medical Image

Computing and Computer-Assisted Intervention – MICCAI 2015 9349, 556–64 DOI: https://doi.org/10.1007/978-3-319-24553-9_68.

9. Clark K, Vendt B, Smith K, Freymann J, Kirby J, Koppel P, et al. The Cancer Imaging Archive (TCIA): Maintaining and Operating a Public Information Repository. J Digit Imaging. 2013 Dec;26(6):1045-57.

10. He K, Zhang X, Ren S, Sun J. Deep residual learning for image recognition. 2016 IEEE Conference on Computer Vision and Pattern Recognition (CVPR), 770–8.

11. Russakovsky O, Deng J, Su H, Krause J, Satheesh S, Ma S, et al. ImageNet Large Scale Visual Recognition Challenge. Int J Comput Vis. 2015 Dec;115(3):211-52.

12. Ronneberger O, Fischer P, Brox T. U-net: Convolutional networks for biomedical image segmentation. Proceedings of 18th International Conference on Medical Image Computing and Computer-Assisted Intervention. 2015 234–41.

13. Pedregosa F, Varoquaux G, Gramfort A, Michel V, Thirion B, Grisel O, et al. Scikit-learn: Machine learning in Python. Journal of Machine Learning Research. 2011 12: 2825–30.

14. Abraham, N. & Khan, N. M. A novel focal tversky loss function with improved attention u-net for lesion segmentation. 2019 IEEE 16th International Symposium on Biomedical Imaging, 683-7, DOI:10.1109/ISBI.2019.8759329 (2019).

15. Tversky A. Features of similarity. Psychological review. 1977 84:327–52.

16. Tiulpin A. Solt: Streaming over lightweight transformations, DOI: 10.5281/zenodo.3702819 (2019).

17. Kingma DP, Ba J. Adam: A method for stochastic optimization. 2014 arXiv:1412.6980.

18. Isosalo A, Inkinen S, Heino H, Turunen T, Nieminen M. MammogramAnnotationTool: Markup tool for breast tissue abnormality annotation. Software Impacts, 2024 19.

19. Lin TY, Dollar P, Girshick R, He K, Hariharan B, Belongie S. Feature pyramid networks for object detection. Proceedings of the IEEE conference on computer vision and pattern recognition, 2017 2117–25.

20. Dai S, Zhu Y, Jiang X, Yu F, Lin J, Yang D. TD-Net: Trans-Deformer network for automatic pancreas segmentation. Neurocomputing. 2023 Jan;517:279-93.

21. Bhinder B, Gilvary C, Madhukar NS, Elemento O. Artificial Intelligence in Cancer Research and Precision Medicine. Cancer Discovery. 2021 Apr 1;11(4):900-15.

22. Yan Y, Zhang D. Multi-scale U-like network with attention mechanism for automatic pancreas segmentation. PLoS ONE. 2021 May 27;16(5):e0252287.

23. Li J, Lin X, Che H, Li H, Qian X. Pancreas segmentation with probabilistic map guided bi-directional recurrent UNet. Phys Med Biol. 2021 Jun 7;66(11):115010.

24. Lim S, Kim YJ, Park Y, Kim D, Kim KG, Lee D. Automated pancreas segmentation and volumetry using deep neural network on computed tomography. Sci Rep. 2022 Mar 8;12(4075).

25. Lee ES. Imaging diagnosis of pancreatic cancer: A state-of-the-art review. WJG. 2014;20(24):7864-77.

26. Shen C, Roth HR, Hayashi Y, Oda M, Miyamoto T, Sato G, et al. A cascaded fully convolutional network framework for dilated pancreatic duct segmentation. Int J CARS. 2022 Feb;17(2):343-54.

27. Janssen BV, Verhoef S, Wesdorp NJ, Huiskens J, de Boer OJ, Marquering H, et al. Imaging-based Machine-learning Models to Predict Clinical Outcomes and Identify Biomarkers in Pancreatic Cancer. Annals of Surgery. 2022 Mar;275(3):560-7.

28. Chen P, Wu T, Wang P, Chang D, Liu K, Wu M, et al. Pancreatic Cancer Detection on CT Scans with Deep Learning: A Nationwide Population-based Study. Radiology. 2023 Jan;306(1):172-82.

29. Kavur AE, Gezer NS, Baris M, Sahin Y, Ozkan S, Baydar B, et al. Comparison of semi-automatic and deep learning-based automatic methods for liver segmentation in living liver transplant donors. Diagn Interv Radiol. 2020 Jan 2;26(1):11-21.

30. Hameed BS, Krishnan UM. Artificial Intelligence-Driven Diagnosis of Pancreatic Cancer. Cancers. 2022 Oct 31;14(21):5382.

31. Kumar Y, Koul A, Singla R, Ijaz MF. Artificial intelligence in disease diagnosis: a systematic literature review, synthesizing framework and future research agenda. J Ambient Intell Human Comput. 2023 Jul;14(7):8459-86.

Click here to access/download;Figure;Fig 1.tiff

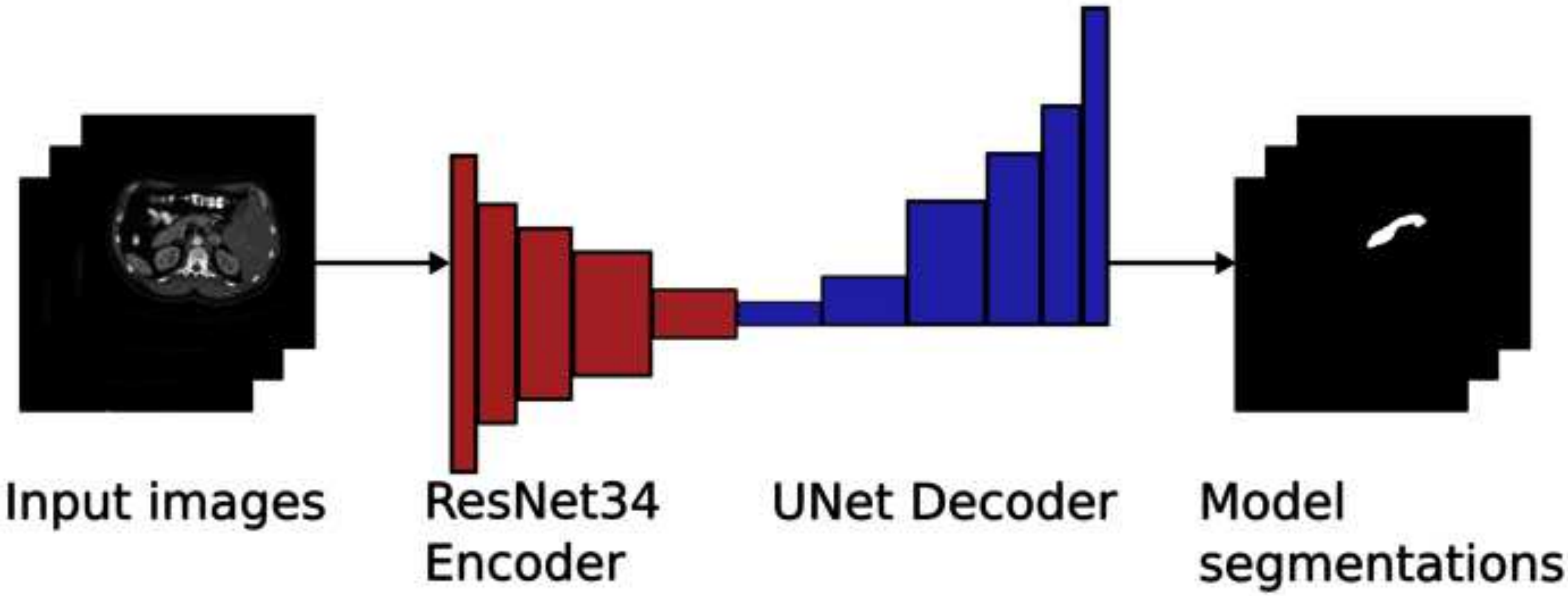

Figure 2

Click here to access/download;Figure;Fig 2.tiff

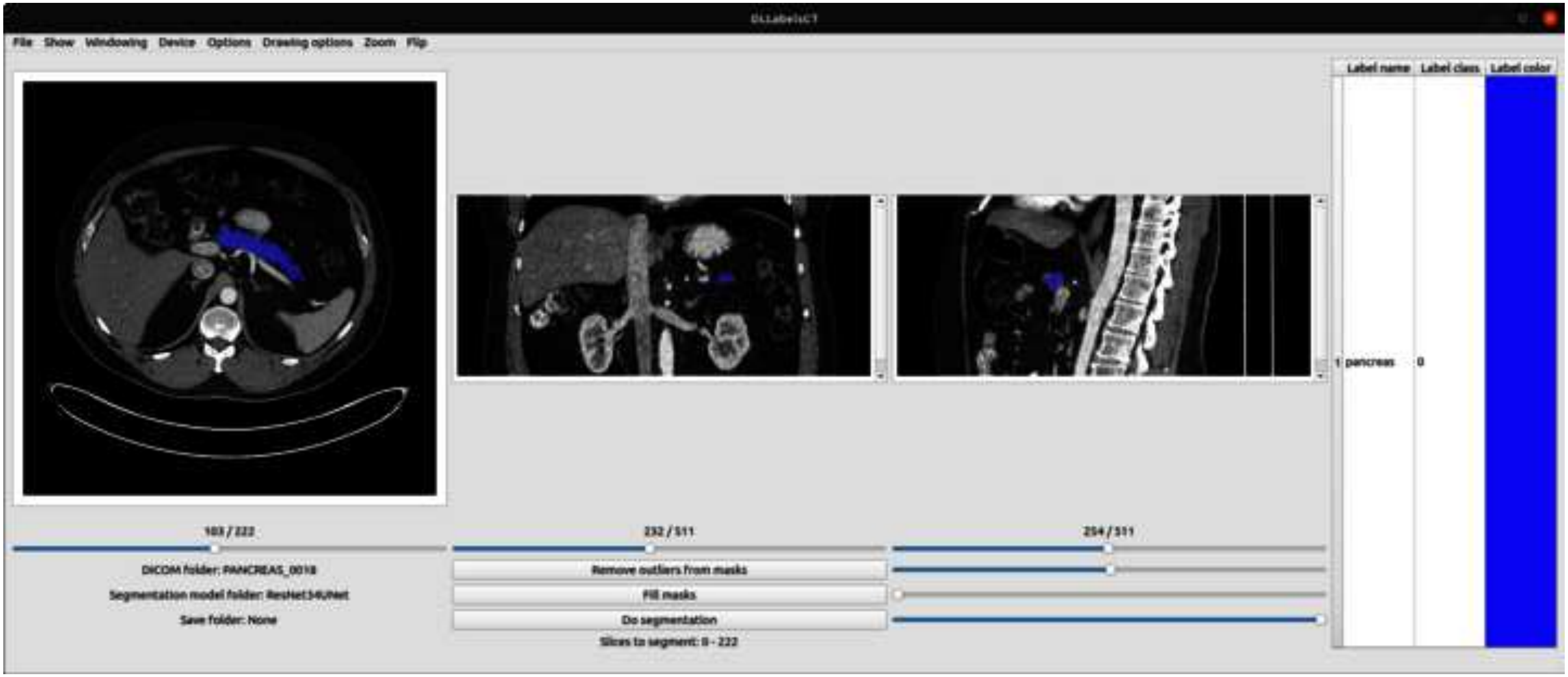

Figure 3

Click here to access/download;Figure;Fig 3.tiff

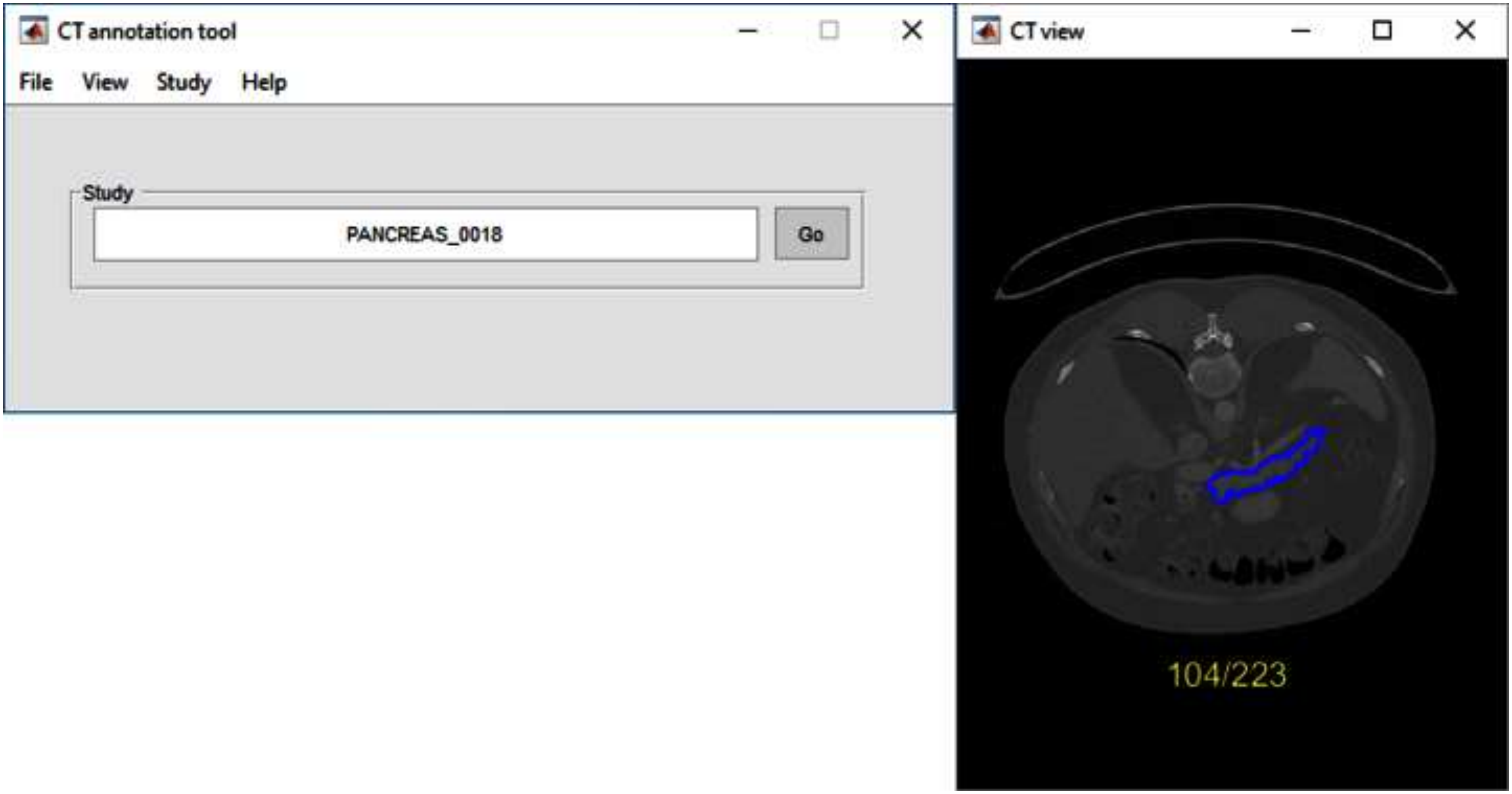

Figure 4

Click here to access/download;Figure;Fig 4.tiff

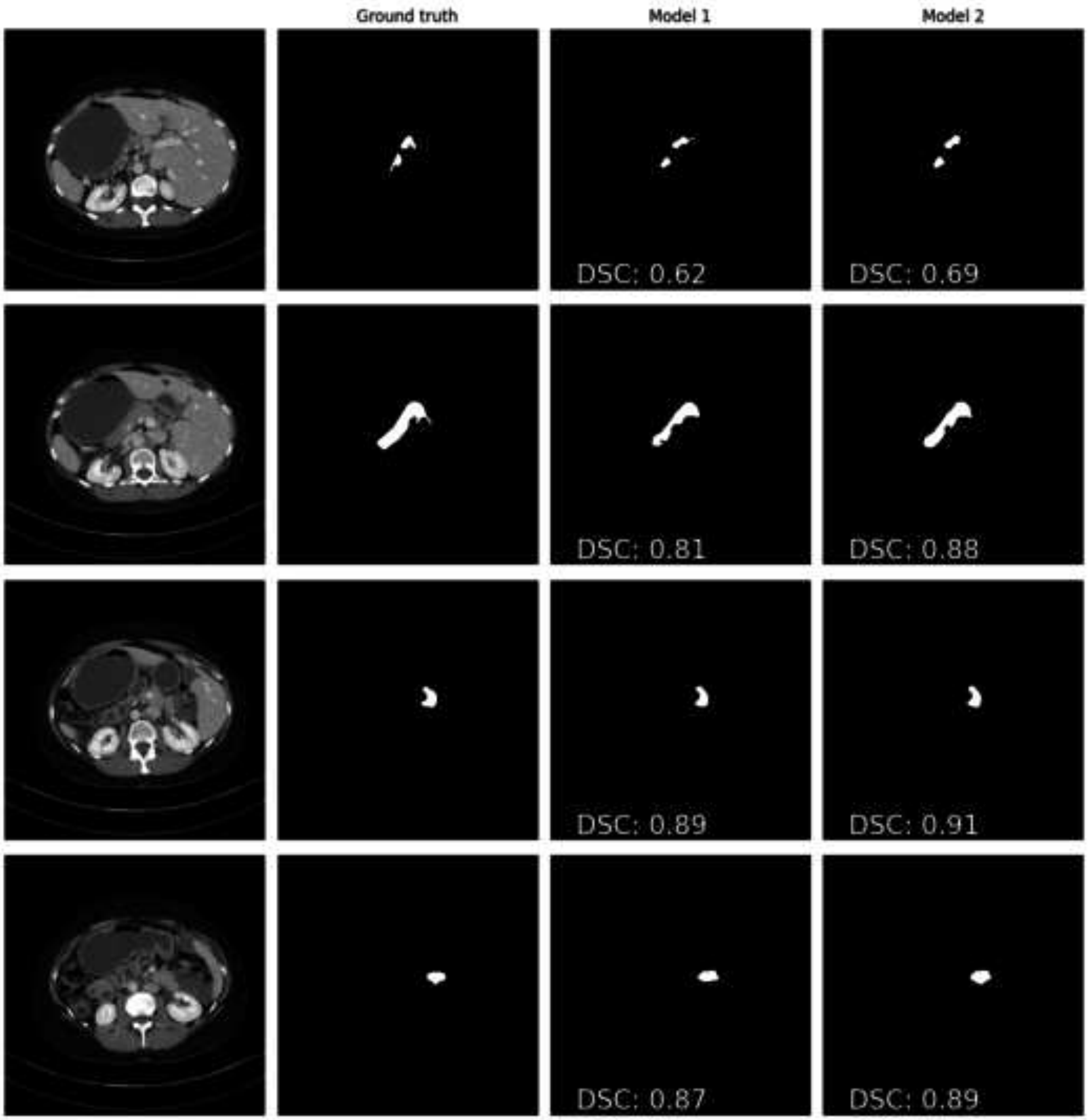

Supplement: Attachment — Submitted filename: PONE-D-24-21753_reviewer-.pdf [file pone.0313126.s001.pdf]
