# Supplementary material for: DLLabelsCT: Annotation tool using deep transfer learning to assist in creating new datasets from abdominal computed tomography scans, case study: Pancreas
Source: PLoS One. 2024 Dec 3;19(12):e0313126. doi: 10.1371/journal.pone.0313126 (PMC11614254; doi:10.1371/journal.pone.0313126)
Supplement: Attachment — Submitted filename: Response to Reviewers.docx [file pone.0313126.s002.docx]

Dr. H.K. Aljobouri

Academic Editor

PLOS ONE

Re: Resubmission after revision: Manuscript ID: PONE-D-24-21753

Dear Dr. Aljobouri,

We thank you for the opportunity to revise and resubmit our manuscript entitled “DLLabelsCT: Annotation tool using deep transfer learning to assist in creating new datasets from abdominal computed tomography scans, case study: pancreas” for PLOS ONE. We appreciate the scientific criticism and advice provided to improve our manuscript. We have now thoroughly addressed the criticism and hope that our changes meet your and the reviewers’ concerns in a satisfactory manner.

Please, below you will find our responses to your comments in your letter dated 07/08/2024.

Sincerely,

Henrik Mustonen

Corresponding author

[henrik.mustonen@oulu.fi](mailto:henrik.mustonen@oulu.fi)

Research Unit of Health Sciences and Technology, Faculty of Medicine, University of Oulu, Oulu, Finland

Academic editor’s comments:

Journal Requirements:

1. When submitting your revision, we need you to address these additional requirements.

Please ensure that your manuscript meets PLOS ONE's style requirements, including those for file naming. The PLOS ONE style templates can be found at
 <https://journals.plos.org/plosone/s/file?id=wjVg/PLOSOne_formatting_sample_main_body.pdf> and
 <https://journals.plos.org/plosone/s/file?id=ba62/PLOSOne_formatting_sample_title_authors_affiliations.pdf>

**Author response:** Thank you for your comment, the manuscript will be changed to meet these requirements.

2. Please note that PLOS ONE has specific guidelines on code sharing for submissions in which author-generated code underpins the findings in the manuscript. In these cases, we expect all author-generated code to be made available without restrictions upon publication of the work. Please review our guidelines at <https://journals.plos.org/plosone/s/materials-and-software-sharing#loc-sharing-code> and ensure that your code is shared in a way that follows best practice and facilitates reproducibility and reuse.

**Author response:** We agree with this, and have already made the annotation tool’s code open-source and available in https://github.com/MIPT-Oulu/DLLabelsCT

3. We note that the grant information you provided in the ‘Funding Information’ and ‘Financial Disclosure’ sections do not match.

When you resubmit, please ensure that you provide the correct grant numbers for the awards you received for your study in the ‘Funding Information’ section.

**Author response:** There seems to have been a misunderstanding during the initial	submission, this will be fixed in the resubmission.

Additional Editor Comments:

The conclusion section must be added and separated from the discussion section.
**Author response**: This will be done in the resubmission.

Reviewer #1: This study introduces an annotation tool utilizing convolutional neural networks to enhance image analysis in medical research called DLLabelsCT. It aims to significantly reduce the time and labor needed for annotating medical images. The authors used pancreas CT scans as data. The study demonstrates DLLabelsCT's speed and accuracy, making it valuable for large datasets and adaptable for different organs. The authors compared their results with a modified MATLAB-based CTAnnotationTool, showing that DLLabelsCT is accurate, time-efficient, and resource-saving. Additionally, the authors provided all necessary libraries and packages, ensuring reproducibility for future research. This study is very impressive. I have a couple of comments:

• The authors mentioned that in literature there are few annotation tools that support DL methods (Introduction second paragraph). It would be helpful to include more information about the existing methods, such as the models they used, their performance, and the size and type of datasets they trained on.

**Author response**: It is true that our article does not give much information about other annotation tools with deep learning capabilities. However, the articles about those tools focused more on the tool itself, instead of the deep learning model and their performance, meaning that such a comparison is not possible. Our manuscript focuses more on showing that a deep learning model can be trained to assist in annotating with a small open access dataset (Pancreas-CT in our case).

Following changes were made to text, page 3 introduction section: *“However, Philbrick et al. concentrated primarily on the mechanical aspects of the annotation tool, rather than illustrating how a deep learning model could be trained to aid in the annotation process.”*

• A flow chart of the whole process would be very useful, especially for understanding the datasets used. For instance, the authors have training, validation, and testing datasets consisting of different sources. Creating a visualization that shows which data was used for training before medical professionals' review, and which dataset was used afterward, would make the study much clearer.

**Author response:** Thank you for your comment, we agree that such a flow chart would be useful and have added it into the manuscript as Figure 2

• The comparison with CTAnnotationTool regarding time and resources is very useful (Table 4, and 5). However, it would be beneficial to include performance metrics for CTAnnotationTool such as recall and precision. This would provide a clearer demonstration of DLLabelsCT's superior speed and also performance compared to CTAnnotationTool.

**Author response:** We agree with the referee that including metrics for the CTAnnotationTool would be demonstrative. However, since the CTAnnotationTool lacks machine learning capabilities, it is not feasible to provide such performance metrics.

Reviewer #2: dear authers
 in the section (materials) you mentioneed the chosen image size of 512 by 512, it is advised to provide in short the logic behind this size.

**Author response:** Thank you for your comment, this image size is the size of the CT –scans, there were no other options.

in the section (training dataset) the scanning systems mentioned in the study have different manufacturer, what effect does this has on the aqcuired data and how does this eventually affect the model?

**Author response:** Referee pointed out an excellent detail. Generally, CT scans taken with different CT scanners provide some information to the model about how varied the imaging can be, with different pixel spacings and slight variations in grayscale values, etc. This eventually leads to the model better responding to those variations, increasing the segmentation quality.

in the section (validation dataset) the dataset from Oulu university was explained stating that 218 scans were cancerous, what methods of diagnosis were used?

**Author response:** Thank you for your comment, all scans from the patients with cancer imaging were later treated with surgery, and the diagnosis was based on the pathological examination of surgical specimens

in the section (model training) it is mentioned that augmentation was used, how did this change the results?

**Author response:** This is a good comment, augmentations have been proven to be an effective way to reduce model overfitting during training, especially on smaller datasets. This means that the model responds better to variations in the dataset and the model provides better quality segmentations on images that it has not seen.

in the same section it is specified that a number of non pancrease containing slices were eleminated to enhance the time factor, could you please specify the amount in time redcuction if possible, what was the time consumed before eliminating the slices and what time was consumed after?

**Author response:** Thank you for your comment, the datasets (Pancreas-CT and Oulu validation dataset) contained a total of 115,371 axial slices. The initial training dataset contained 18,942 axial slices and the model training took 24 hours. If all of those 115,371 axial slices were used during training, the model training would take around a week. This would’ve been too long for us, which means that the number of slices had to be reduced.
