# Supplementary material for: DLLabelsCT: Annotation tool using deep transfer learning to assist in creating new datasets from abdominal computed tomography scans, case study: Pancreas
Source: PLoS One. 2024 Dec 3;19(12):e0313126. doi: 10.1371/journal.pone.0313126 (PMC11614254; doi:10.1371/journal.pone.0313126)
Supplement: Attachment — Submitted filename: Response to Reviewers.docx [file pone.0313126.s003.docx]

Dr. H.K. Aljobouri

Academic Editor

PLOS ONE

Re: Resubmission after revision: Manuscript ID: PONE-D-24-21753R1

Dear Dr. Aljobouri,

We thank you for the opportunity to revise and resubmit our manuscript entitled “DLLabelsCT: Annotation tool using deep transfer learning to assist in creating new datasets from abdominal computed tomography scans, case study: pancreas” for PLOS ONE. We appreciate advice provided to improve our manuscript. We have now thoroughly addressed the criticism and hope that our changes meet your concerns in a satisfactory manner.

Please, below you will find our responses to your comments in your letter dated 02/10/2024.

Sincerely,

Henrik Mustonen

Corresponding author

[henrik.mustonen@oulu.fi](mailto:henrik.mustonen@oulu.fi)

Research Unit of Health Sciences and Technology, Faculty of Medicine, University of Oulu, Oulu, Finland

Academic editor’s comments:

Journal Requirements:

Please review your reference list to ensure that it is complete and correct. If you have cited papers that have been retracted, please include the rationale for doing so in the manuscript text, or remove these references and replace them with relevant current references. Any changes to the reference list should be mentioned in the rebuttal letter that accompanies your revised manuscript. If you need to cite a retracted article, indicate the article’s retracted status in the References list and also include a citation and full reference for the retraction notice.

**Author response:** We have gone through the references and have not found any papers that have been retracted. We have updated the References list to be more consistent and added a few more references. These references are number [3], [14] and [20] in the manuscript.

Additional Editor Comments:
The number of references is very small, we prefer to add more.

**Author response:** We disagree with the comment regarding the low number of references. The 31 references in the original research article are adequate, and adding more references does not diminish the impact of this article. However, as the editor has requested more references, we have added three additional references to the manuscript.
